# Appointment

**Published:** 1862-03

**Authors:** 


APPOINTMENT.—E. P. GREY, M. D., of Buffalo, Surgeon to the —— Regiment, N. Y. S. V.

